# Luteimonas salinilitoris sp. nov., isolated from the shore soil of saline lake in Tibet of China

**DOI:** 10.1099/ijsem.0.006630

**Published:** 2025-01-06

**Authors:** Xuan Zhang, You-Jun Liao, Zi-Xuan Liu, Rui Wang, Hong-Can Liu, Yan-Yan Zheng, Yu-Guang Zhou, Ya-Jing Yu, Lu Xue, Ai-Hua Li

**Affiliations:** 1 School of Biotechnology and Food Science, Tianjin University of Commerce, Tianjin, PR China; 2 China General Microbiological Culture Collection Center (CGMCC), Institute of Microbiology, Chinese Academy of Sciences, Beijing 100101, PR China; 3 Tianjin Institute of Industrial Biotechnology, Chinese Academy of Sciences, Tianjin 300308, PR China; 4 Tibet Plateau Key Laboratory of Mycology, Tibet Plateau Institute of Biology, Lhasa, Tibet 850001, PR China

**Keywords:** *Luteimonas salinilitoris *sp. nov., saline lake, soil, Tibet

## Abstract

Five aerobic, Gram-stain-negative bacterial strains, designated as C3-2-a3^T^, B3-2-R+30, C3-2-a4, C3-2-M3 and C3-2-M8, were isolated from the coastal soil of LungmuCo Lake in the Tibet Autonomous Region, PR China. Phylogenetic analyses based on 16S rRNA genes and genomes indicated that these isolates belonged to the genus *Luteimonas* and showed a high similarity to *Luteimonas suaedae* LNNU 24178^T^ (99.01%), *Luteimonas endophytica* RD2P54^T^ (98.80%) and *Luteimonas salinisoli* SJ-92^T^ (97.67%). The average nucleotide identity (ANI) and digital DNA–DNA hybridization (dDDH) values between strain C3-2-a3^T^ and related reference strains *Luteimonas suaedae* LNNU 24178^T^, *Luteimonas endophytica* RD2P54^T^ and *Luteimonas salinisoli* SJ-92^T^ were 91.89, 83.11 and 83.86% and 46.90, 26.90 and 28.20%, respectively. All values were below the thresholds for delineating species, supporting their classification as novel species of the genus *Luteimonas*. The genomic DNA G+C content of strains C3-2-a3^T^ was 68.39%. The major polar lipids were diphosphatidylglycerol, phosphatidylglycerol, phosphatidylethanolamine and two unidentified phospholipids. The predominant respiratory quinone was ubiquinone-8 (Q-8), aligning with the characteristics of members of the genus *Luteimonas*. The major fatty acids (>10.0%) of strain C3-2-a3^T^ were identified as iso-C_11 :  0_, iso-C_15 :  0_, iso-C_16 : 0_ and iso-C_17 : 1_* ω*9*c*. Based on the results of phenotypic, physiological, chemotaxonomic and genotypic characterizations, we propose that the isolates represent a novel species of genus *Luteimonas*, for which the name *Luteimonas salinilitoris s*p. nov is proposed. The type strain is C3-2-a3^T^ (=CGMCC 1.14507^T^=KCTC 8642^T^).

## Introduction

The genus *Luteimonas* within the family *Lysobacteraceae* and class *Gammaproteobacteria* was first proposed by Finkmann *et al.* [1], with *Luteimonas mephitis* as the type species. It is a group of Gram-stain-negative, aerobic, rod-shaped bacteria with ubiquinone-8 (Q-8) as the predominant ubiquinone and iso-C_15 : 0_ as the major fatty acid. At the time of writing, genus *Luteimonas* comprises 34 species with validly published and correct names (https://lpsn.dsmz.de/genus/luteimonas), isolated from different habitats such as sediment [2,3], fresh water [4], rectal contents of Tibetan Plateau pika [5], food waste [6], cucumber leaf [7] and soil [8,12].

LungmuCo Lake is a saline lake in the Ali region of Tibet, with an altitude of 5030 m and an annual average temperature ranging from −6 to 9 ℃. The lake is abundant in minerals, and there are numerous ancient embankments along its shores, showcasing the retreat of the saline lake. Due to the lake’s unique geographical and chemical characteristics, as well as its remoteness from human interference, the microbial resources inhabiting the lake are also distinctive [13]. For this reason, microbial resource collection and biodiversity investigation were carried out at LungmuCo Lake.

In this study, we isolated five strains (C3-2-a3^T^, B3-2-R+30, C3-2-a4, C3-2-M3 and C3-2-M8) that shared the same 16S rRNA gene sequence and belong to the genus *Luteimonas,* based on the 16S rRNA gene sequences analysis in the GenBank database or EzBioCloud server. A polyphasic taxonomy was conducted for two of these strains (C3-2-a3^T^ and B3-2-R+30) to verify their taxonomic status, and we hereby propose a novel *Luteimonas* species, for which the name *Luteimonas salinilitoris* sp. nov. is suggested.

## Methods

### Ecology and isolation

The soil samples used for strain isolation were collected from the soil along the coast of LungmuCo Lake, located in Ritu County of Ali region, Tibet Autonomous Region, PR China (coordinates: 34°33’ N/80°23’ E). Strains were isolated using a traditional separation method: 10 g of soil was weighed into 100 ml of sterilized water, followed by a tenfold dilution and thorough mixing of the solution under aseptic conditions. Finally, 200 µl of the mixed solution from a suitable gradient of homogeneous solution (with dilution gradient of 10^−3^, 10^−4^ and 10^−5^) were spread on various types of plate culture media (marine 2216, R2A, R2A plus 0.5% NaCl). After 2 weeks of culture at 15 ℃, we picked out single colonies and purified them further. All purified strains were preserved by lyophilization and liquid nitrogen.

### 16S rRNA gene phylogeny

The 16S rRNA gene was amplified for strains C3-2-a3^T^, B3-2-R+30, C3-2-a4, C3-2-M3 and C3-2-M8 by PCR using the universal primers 27F and 1492R. To verify the evolutionary position of these strains, their 16S rRNA gene sequences were aligned with the available 16S rRNA genes of validly published species through the National Center for Biotechnology Information (NCBI) GenBank database using the blast program (https://blast.ncbi.nlm.nih.gov/Blast.cgi) to determine their phylogenetic affiliation. Further phylogenetic analyses were performed using mega 7.0 [14] to construct phylogenetic trees by neighbour-joining, maximum-likelihood and maximum-parsimony methods. The bootstrap values were based on 1000 replicates [15].

### Genome features

For the analysis of genome relatedness, we obtained the whole genomes of strains C3-2-a3^T^ and B3-2-R+30 by the Illumina NovaSeq X plus platform (at Majorbio Science and Technology Ltd, China). The raw sequencing data were detected and analysed using statistical methods; the clean sequencing data were assembled with SOAP *de novo* v2.0 (http://soapdenovo2. sourceforge.net/) [16]. Subsequently, we acquired the whole genome of type strains *Luteimonas salinisoli* SJ-92^T^, *Luteimonas suaedae* LNNU 24178^T^ and *Luteimonas endophytica* RD2P54^T^ from the NCBI GenBank database. We constructed a phylogenetic tree based on the whole genome using Type (Strain) Genome Server (TYGS) online server to further estimate the phylogenetic position of strains C3-2-a3^T^ and B3-2-R+30 [17].

The average nucleotide identity (ANI) between the genomes of these strains was calculated using an ANI Calculator tool in EzBioCloud (https://www.ezbiocloud.net/tools/ani) [18]. Digital DNA–DNA hybridization (dDDH) values between the genomes of strains were computed using Formula 2 of the Genome-to-Genome Distance Calculator (http://ggdc.dsmz.de/ggdc.php) [19]. The DNA G+C content was calculated from the draft genome sequence.

Genome prediction was performed using Glimmer (v3.02; http://ccb.jhu.edu/software/glimmer/index.shtml) and GeneMarks (v4.3; http://topaz.gatech.edu/GeneMark), Barrnap (v0.8; https://github.com/tseemann/barrnap) and tRNA-scan-SE (v2.0; http://trna.ucsc.edu/software/). Among them, Glimmer and GeneMarks were utilized to predict coding sequences, Barrnap was employed to predict rRNA, tRNA-scan-SE was used for tRNA prediction [20].

The gene function was annotated using the eggNOG (evolutionary genealogy of genes: Non-supervised Orthologous Groups, http://eggnogdb.embl.de/#/app/home) and KEGG (Kyoto Encyclopedia of Genes and Genomes, http://www.genome.jp/kegg/) databases [21]. Additionally, the gene cluster involved in secondary metabolites synthesis was predicted by using the antiSMASH (https://dl.secondarymetabolites.org/releases/4.0.2/) database [22].

### Phenotypic and physiological characteristics

Gram staining was performed using the traditional method described by Claus [23]. Cell morphology (grown at 25 ℃ for 2–3 days on MA medium) was examined with a transmission electron microscope. The growth of the strains at different temperatures (4, 10, 15, 20, 25, 30, 35, 40 and 42 °C) was tested by R2A liquid medium for 14 days; tolerance of NaCl (0, 0.5;1.0–18.0%, at 2.0% intervals) and the pH range for growth (5.0–11.0, at intervals of 1.0 pH unit) were assessed using R2A liquid medium for 14 days. Biological buffers were used to adjust the pH of the medium, including 100 mM citric acid/sodium citrate buffer for pH 5.0–6.0, 100 mM Na_2_HPO_4_/NaH_2_PO_4_ buffer for pH 7.0–8.0 and 100 mM NaHCO_3_/Na_2_CO_3_ buffer for pH 9.0–11.0. Cell density in the culture was measured as OD_600_ using a UV/visible spectrophotometer (Lambda 950, Perkin Elmer Co). Oxidase activity was assessed with the Bactident Oxidase Strips, Merck detection kit. Catalase activity was tested by observing bubble production through dripping 5.0% (v /v) H_2_O_2_. To evaluate substrate degradation, MA medium was supplemented with 5.0% casein (skimmed milk), 1.0% cellulose sodium, 1.0% Tween 20, 1.0% Tween 80 and 1.0% soluble starch (w/v), respectively. Additional enzyme activities were examined using API ZYM, API 20NE strips, following the manufacturer’s instructions. Utilization of sole carbon sources was determined using the Biology GEN III MicroPlate system, and acid production was assessed using the API 50CH following the manufacturer’s instructions. Antibiotic sensitivity tests were performed using the disc diffusion method [24] on MA plates with 8 mm-diameter filter-paper discs containing the following antibiotics: fleroxacin (5 µg), lomefloxacin (10 µg), ciprofloxacin (5 µg), penicillin (10 µg), erythromycin (15 µg), chloramphenicol (15 µg), azithromycin (15 µg), clindamycin (2 µg), doxycycline (30 µg), clarithromycin (15 µg), tobramycin (10 µg), vancomycin (30 µg), netilmicin (30 µg), ceftriaxone (30 µg), cefaclor (30 µg), cefazolin (30 µg), cefotaxime (10 µg), ampicillin (10 µg), cefuroxime (30 µg), minocycline (30 µg), rifampicin (5 µg), tetracycline (30 µg), sulfamethoxazole (25 µg)，amikacin (30 µg), ceftazidime (30 µg), cefotaxime (30 µg), cefoperazone (75 µg)，piperacillin (10 µg), oxacillin (1 µg) and nitrofurantoin (300 µg).

### Chemotaxonomic characteristics

For chemotaxonomic analysis, *Luteimonas salinisoli* SJ-92^T^, *Luteimonas suaedae* LNNU 24178^T^ and *Luteimonas endophytica* RD2P54^T^ were used as reference strains based on phylogenetic analysis of the 16S rRNA gene sequence and genome. Cells of strains C3-2-a3^T^ and B3-2-R+30, and reference strains were incubated in MB at 30℃ and harvested at the same physiological age during the initial stationary phase. The freeze-dried cells were obtained through centrifugation and freeze-drying. Polar lipids were extracted via chloroform–methanol and analysed using thin-layer chromatography following the method by Minnikin *et al.* [25]. The TLC plates were sprayed with several specific reagents, including molybdatophosphoric acid, phosphomolybdic acid, ninhydrin and *α*-naphthol/sulphuric acid reagents, respectively, for the detection of total lipids, phospholipids, aminolipids and glycolipids.

Respiratory quinones were extracted through chloroform–methanol and analysed using high-performance liquid chromatography (HPLC 1260, Agilent; YMC, AA12S05-1546WT column), according to the method of Collins *et al.* [26]. For cellular fatty acid analysis, cells of strains C3-2-a3^T^ and B3-2-R+30, as well as the reference strains *Luteimonas salinisoli* SJ-92^T^*, Luteimonas suaedae* LNNU 24178^T^ and *Luteimonas endophytica* RD2P54^T^, were cultured on MA plates at 30℃ for 72 h. Harvesting, saponification, methylation and extraction of cellular fatty acids were performed according to the standard protocol in the MIDI Packard Microbial Identification System. The extraction was analysed by gas chromatography (model 6890 N, Agilent; UItra-2 column) and identified using the Microbial Identification software package version 6.0 with the TSBA 6.0 database [27]. To further differentiate the fatty acid components of these strains, which could not be separated by gas chromatography, a gas chromatography-mass spectrometry instrument (GC MS-QP2010 UItra, SHIMADZU; HP-5MS-30M column) was utilized for fatty acids analysis. The program began at 150 ℃ and was maintained for 3 min, then increased to 280 ℃ at a rate of 2 ℃/min and finally reached 325 ℃ at 30 ℃/min. The inlet temperature was set to 280 ℃.

## Results and conclusion

### Phylogenetic and genome analysis

**Fig. 1.**
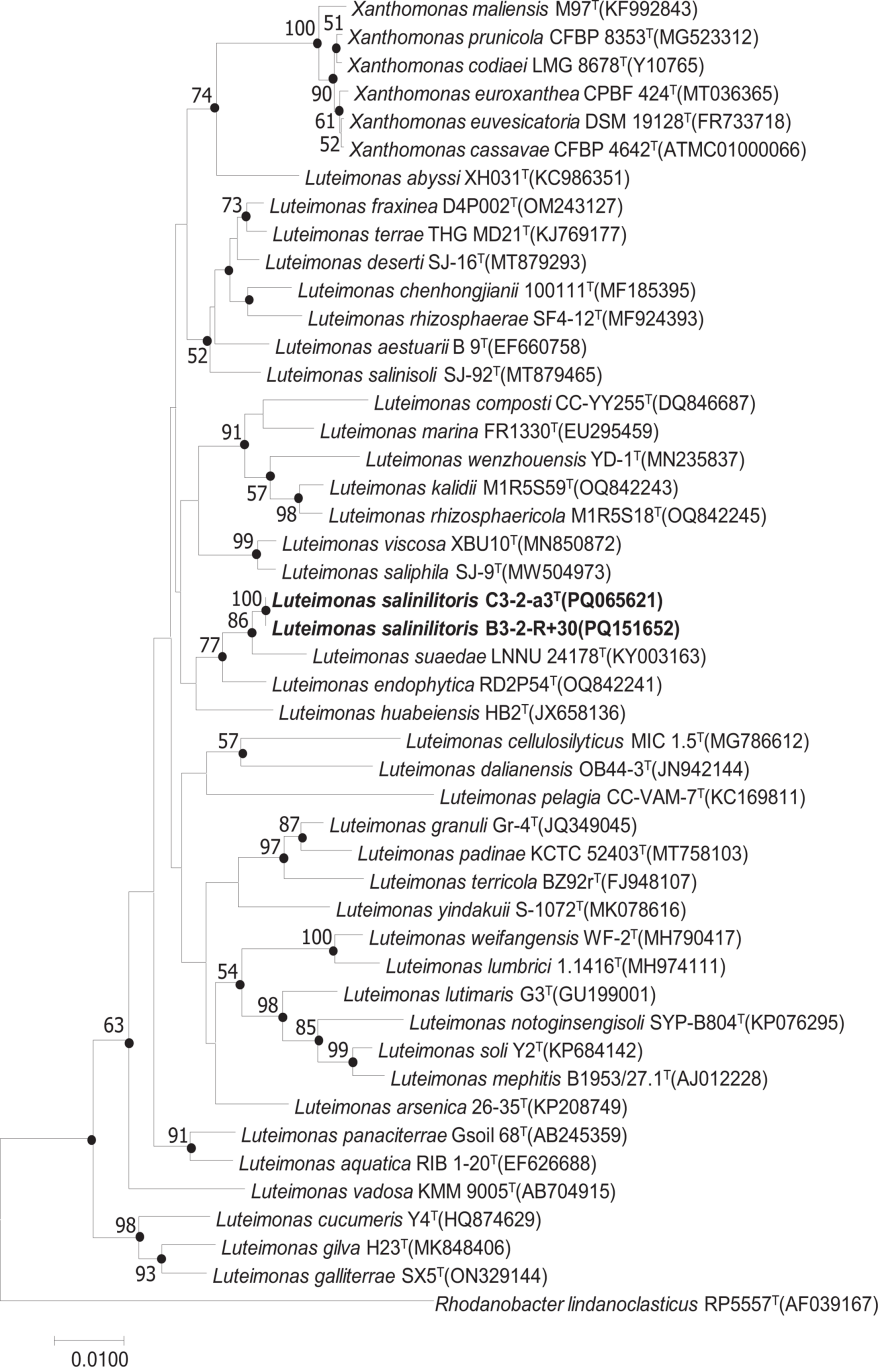
Neighbour-joining phylogenetic tree based on 16S rRNA gene sequences. Bootstrap values >50% are shown at branch points based on 1000 resamplings. The filled circles indicate that the corresponding nodes were recovered in the maximum-likelihood and maximum-parsimony methods. *Vulcaniibacterium tengchongense* DSM 25623^T^ was used as an outgroup.

The almost complete 16S rRNA gene sequences of strains C3-2-a3^T^ (1415 bp) and B3-2-R+30(1403 bp) were acquired and compared with each other and the type strains in the NCBI GenBank database. The alignment results showed that these five strains exhibited 100 similarity of 16S rRNA gene with one another, and they demonstrated high similarity of 16S rRNA sequences with members of the genus *Luteimonas: Luteimonas suaedae* LNNU 24178^T^ (99.01%), *Luteimonas endophytica* RD2P54^T^ (98.80%) and *Luteimonas salinisoli* SJ-92^T^ (97.67%). The 16S rRNA gene sequences of these novel isolates from Sanger and Illumina sequencing were identical. In the neighbour-joining tree C3-2-a3^T^ and B3-2-R+30 formed a distinct clade with *Luteimonas suaedae* LNNU 24178^T^ and *Luteimonas endophytica* RD2P54^T^, and subsequently clustered together with other species in genus *Luteimonas* (Fig. 1). This relationship was also supported in the maximum-likelihood and maximum-parsimony phylogenetic trees. Although strains C3-2-a3^T^ and B3-2-R+30 appeared to be more closely related to species within the genus *Xanthomonas*, such as the type species *X. campestris*, than to the type species of the genus *Luteimonas*, the ANI and dDDH analyses supported their assignment to the genus *Luteimonas*. The ANI and dDDH between strain C3-2-a3^T^ and *Luteimonas mephitis* were 78.46 and 22.30%, respectively, which were higher than those between it and *X. campestris*, which were 74.73 and 20.70%.

**Fig. 2.**
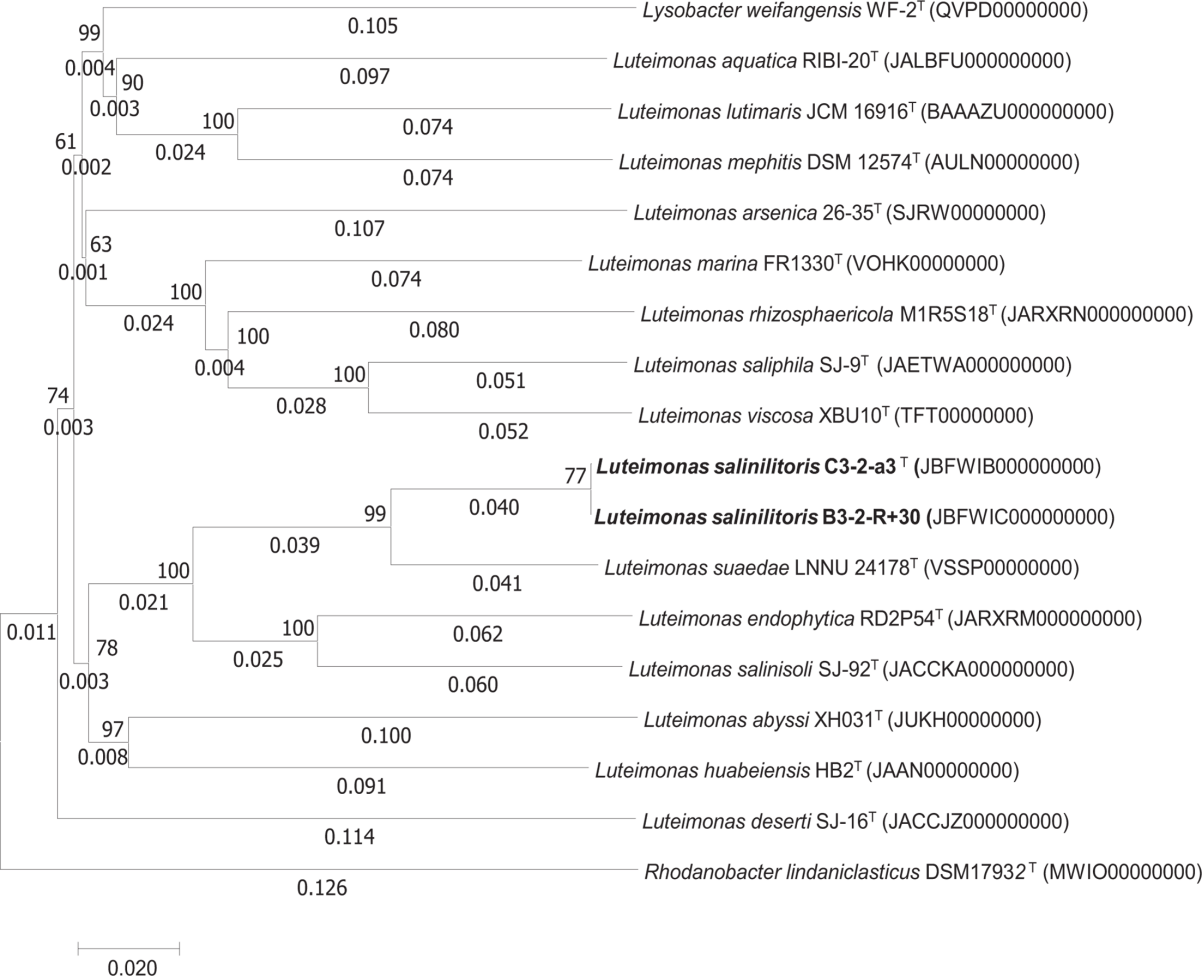
Phylogenomic tree based on the genome sequence of strains C3-2-a3^T^ and B3-2-R+30 on the TYGS. (http://tygs.dsmz.de/). The numbers above branches are GBDP pseudo-bootstrap support values >60% from 100 replications, with an average branch support of 91.1%.

In addition, the phylogenomic tree based on the whole-genome sequences, constructed by the TYGS online server, showed that strains C3-2-a3^T^ and B3-2-R+30 were clustered with *Luteimonas suaedae* LNNU 24178^T^, *Luteimonas endophytica* RD2P54^T^ and *Luteimonas salinisoli* SJ-92^T^, showing their relationship with the species of the genus *Luteimonas* (Fig. 2).

The ANI and dDDH values between strains C3-2-a3^T^ and B3-2-R+30 were 99.75 and 99.50%, respectively, indicating that strains C3-2-a3^T^ and B3-2-R+30 represent the same novel species of *Luteimonas*. The ANI values between strain C3-2-a3^T^ and the reference strains *Luteimonas suaedae* LNNU 24178^T^, *Luteimonas endophytica* RD2P54^T^ and *Luteimonas salinisoli* SJ-92^T^ were 91.89%, 83.11% and 83.86%, respectively. The dDDH values between strain C3-2-a3^T^ and the reference strains *Luteimonas suaedae* LNNU 24178^T^, *Luteimonas endophytica* RD2P54^T^ and *Luteimonas salinisoli* SJ-92^T^ were 46.90, 26.90 and 28.20%, respectively. All values were significantly lower than the proposed threshold values of 95–96% (the ANI values) and 70% (the dDDH values) for describing new species [19,28].

The genomic DNA G+C content of strains C3-2-a3^T^ and B3-2-R+30 were 68.39% and 68.36%, respectively. The genome size of strain C3-2-a3^T^ was 4.65 Mb (Scaffold No: 70; Scaffold N50 : 260586), containing 4132 coding genes, 3 rRNAs (one 16S rRNA, one 23S rRNA and one 5S rRNA) and 43 tRNAs. By comparing COG function, the gene function annotations of strains C3-2-a3^T^ and B3-2-R+30 were categorized into 24 COG functional categories, which fell into the following three categories: information storage and processing, cellular processes and signalling metabolism. The result of the KEGG functional comparison indicated that the function of the strains was annotated as metabolism, human diseases, organismal systems, environmental information processing, genetic information processing and cellular processes, encompassing six pathways at level 1.

Genome mining analysis using the antiSMASH database revealed that strains C3-2-a3^T^ and B3-2-R+30 contained gene clusters associated with bacteriocin, arylpolyenes, resorcinol and nonribosomal peptide synthetase, indicating their potential application in the field of biosynthesis.

### Morphological and physiological property

**Fig. 3.**
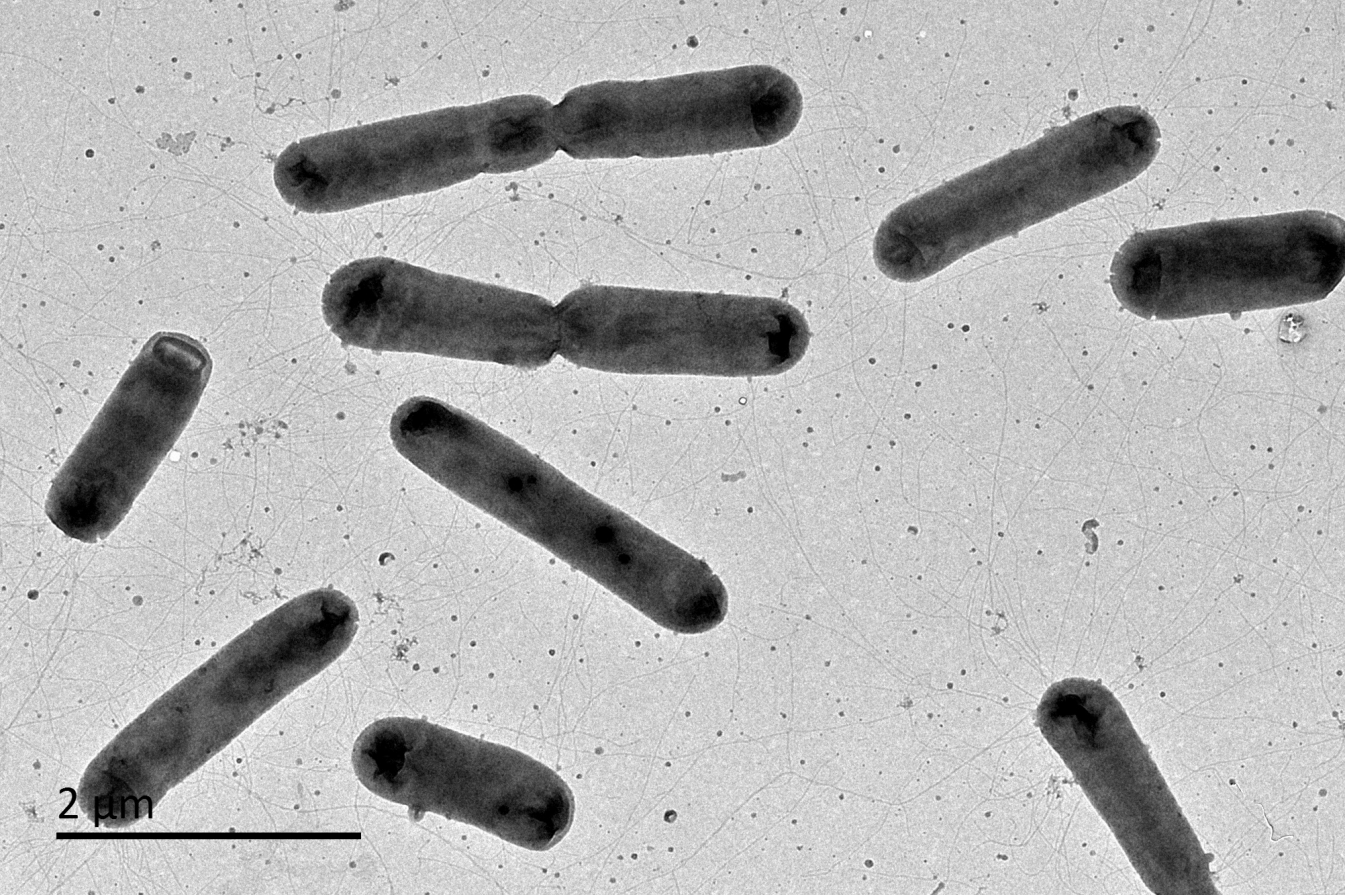
Transmission electron micrograph of strain C3-2-a3^T^ cultivated on the MA plate at 25 ℃ for 48–72 h.

Cells of strain C3-2-a3^T^ were Gram-stain-negative, aerobic, 0.50–0.53 µm in width and 2.54–3.96 µm in length (Fig. 3). The colonies of strains C3-2-a3^T^ were yellow, circular and convex after being cultured on MA solid medium at 30℃ for 3 days. Strains were able to grow at 4–35℃ (optimum,30℃), 0–8.0% NaCl (optimum, 6.0%), pH 6.0–10.0 (optimum, pH 7.0). Cells of strain C3-2-a3^T^ were oxidase-positive and catalase-negative. Additionally, the result of API ZYM showed that strains C3-2-a3^T^ and B3-2-R+30 were positive for alkaline phosphatase, esterase (C4), lipid esterase (C8), leucine arylamidase, valine arylamidase, cystine arylamidase, trypsin, chymotrypsin, acid phosphatase, naphthol-AS-BI-phosphohydrolase, *α*-Galactosidase, *β*-Galactosidase, α-Glucosidase, *β*-Glucosidase, *N*-acetyl-*β*-glucosaminidase and α-Mannosidase, but negative for lipase (C14). In the API 20NE system, strains C3-2-a3^T^ and B3-2-R+30 were positive for aesculin and gelatin hydrolysis, but they did not produce indole and did not ferment glucose, and they were negative for nitrate reduction and arginine dihydrolase hydrolysis. Through the API 50CH system, strains could produce acids from aesculin citrate. The result of the Biology GEN III MicroStation system indicated that the substrates usable by the strains as the sole carbon source included dextrin, d-maltose, d-trehalose, d-cellobiose, gentiobiose, turanose, d-raffinose, *α*-d-lactose, melibiose, d-salicylin, *N*-acetyl-d-glucosamine, *N*-acetyl-d-galactosamine, *α*-d-glucose, d-mannose, d-galactose, l-fucose, inositol, gelatin, l-glutamic acid, *β*-methyl-d-glucoside, d-fructose, d-fucose, Tween 40, *β*-hydroxy-d, l-butyric acid, *α*-butanoic acid, acetoacetate and propionic acid (Table 1). Based on these phenotypic characteristics, strains C3-2-a3^T^ and B3-2-R+30 can be discriminated from the close relatives by their higher optimum growth NaCl concentration. Additionally, both strains can utilize melibiose and inositol as the sole carbon source, whereas other reference strains cannot use these two substances as the sole carbon sources. Strains C3-2-a3^T^ and B3-2-R+30 were both sensitive to azithromycin and ceftazidime; notably, C3-2-a3^T^ was also sensitive to sulfamethoxazole and piperacillin. Detailed differential phenotypic and physiological characteristics of strains C3-2-a3^T^ and B3-2-R+30 with the reference strains are listed in Table 1.

**Table 1.** Differential characteristics between C3-2-a3^T^, B3-2-R+30 and the type strains of closely related species of the genus *Luteimonas* Strains:1, C3-2-a3^T^; 2, B3-2-R+30; 3, *L. salinisoli* SJ-92^T^; 4, *L. suaedae* LNNU 24178^T^; 5, *L. endophytica* RD2P54^T^. +, positive reaction; -, negative reaction; nd, not determined.

Characteristics	1	2	3	4	5
Temperature range for growth (optimal, °C)	4–35 (30)	4–35 (30)	10–35 (30)	15–35 (30)	15–45 (30)
pH range for growth (optimal)	6.0–10.0 (7.0)	6.0–10.0 (7.0)	7.0–11.0 (8.0)	7.0–10.0 (7.0)	6.0–9.0 (7.0)
NaCl tolerance (optimal, %, w/v)	0–8.0 (6.0)	0–8.0 (6.0)	0–8.0 (2.0)	0–7.0 (0–2.0)	0–9.0 (1.0)
Nitrate reduction	−	−	−	−	+
Gelatin hydrolysis	+	+	+	−	+
Assimilation of (Biology GEN III)					
d-Raffinose	w	+	−	w	−
*α*-d-Lactose	+	+	−	−	−
Melibiose	+	+	−	−	−
d-Salicylin	+	+	w	−	−
d-Galactose	+	+	−	w	−
l-Fucose	+	+	w	w	−
Inositol	w	+	−	−	−
l-Serine	−	−	w	+	−
l-Malic acid	−	−	−	+	−
Bromosuccinic acid	−	−	−	+	−
Formic acid	−	−	−	+	−
Enzyme activity (API ZYM):					
*α*-Galactosidase	+	+	−	+	−
*β*-Galactosidase	+	+	+	+	−
*α*-Glucosidase	+	+	−	+	+
*α*-Mannosidase	+	+	−	+	−

### Chemotaxonomic property

**Fig. 4.**
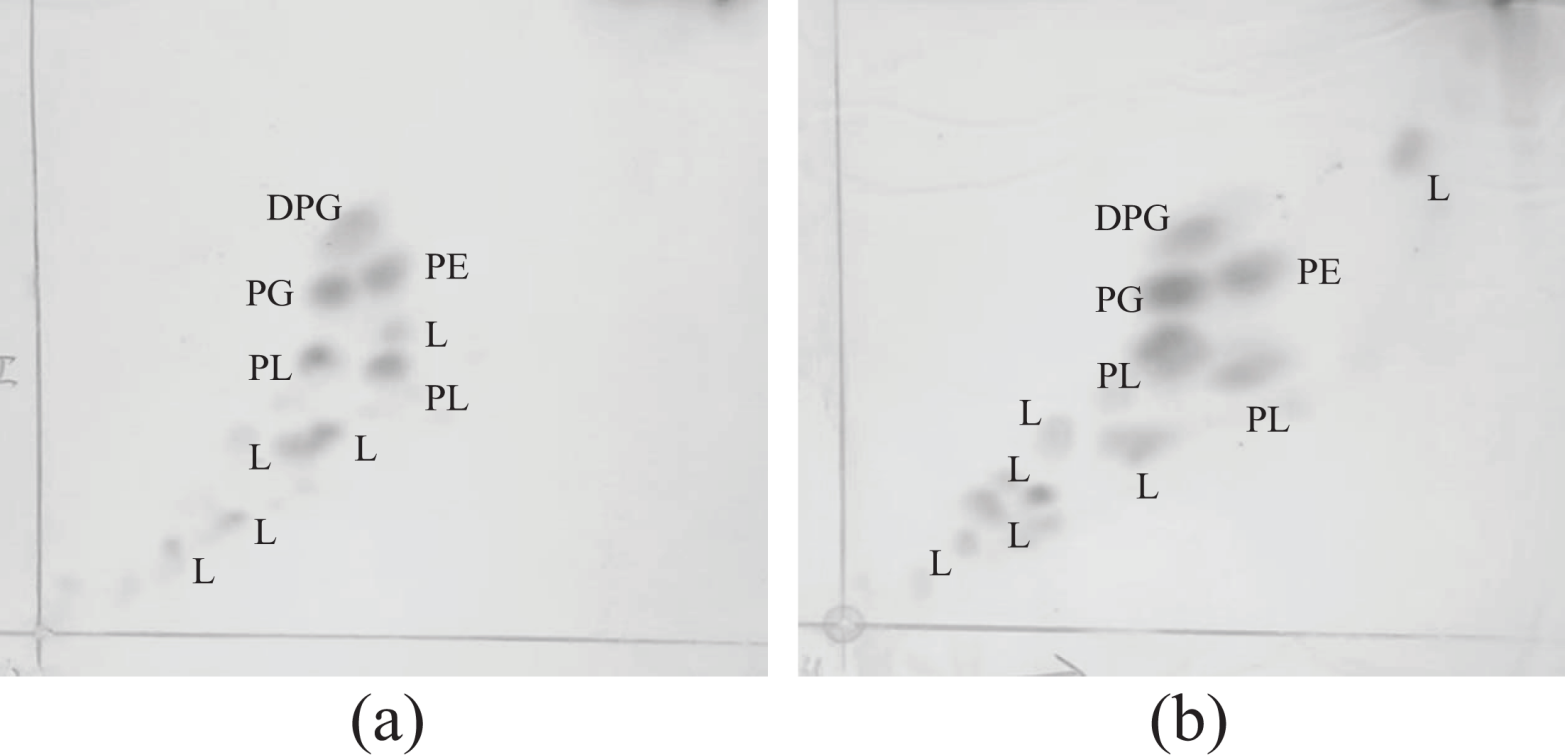
Two-dimensional TLC of the polar lipids of strains C3-2-a3^T^ (**a**) and B3-2-R+30 (**b**) stained with molybdophosphoric acid. DPG, diphosphatidylglycerol; PG, phosphatidylglycerol; PE，phosphatidylethanolamine; PL, unidentified phospholipid; L, unidentified lipid.

**Fig. 5.**
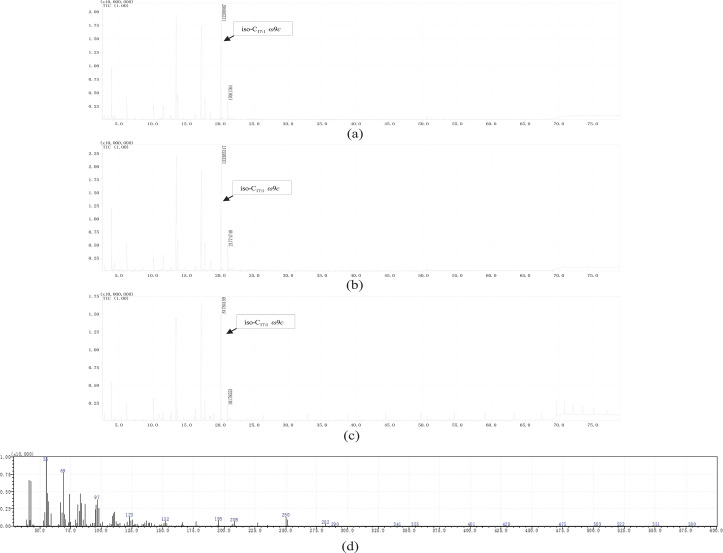
The GC-MS detection of the fatty acid composition summed feature 9 (iso-C_17 : 1_* ω*9*c* / C_16 : 0_ 10-methyl) of the of strains C3-2-a3^T^, B3-2-R+30 and reference strain *Luteimonas suaedae* LNNU 24178^T^. The iso-C_17 : 1_* ω*9*c* in strain C3-2-a3^T^ (**a**), strain B3-2-R+30 (**b**) and *Luteimonas suaedae* LNNU 24178^T^ (**c**) were shown as a peak with RT 19.990 min. The mass spectrum of iso-C_17 : 1_* ω*9*c* in strain C3-2-a3^T^ is shown in (**d**).

The polar lipid profiles of strains C3-2-a3^T^ and B3-2-R+30 indicated that diphosphatidylglycerol, phosphatidylglycerol, phosphatidylethanolamine and two unidentified phospholipids were the major lipids (Fig. 4). The predominant respiratory quinone detected in strains C3-2-a3^T^ and B3-2-R+30 was Q-8, which was the characteristic of members of the genus *Luteimonas*. The cellular fatty acid profiles of strains C3-2-a3^T^, B3-2-R+30 and related reference strains are listed in Table 2. The major fatty acids (>10.0%) of strains C3-2-a3^T^ and B3-2-R+30 identified by GC were shown as iso-C_11  :  0_ (11.2%, 12.0%), iso-C_15  :  0_ (17.1%, 16.9%), iso-C_16  :  0_ (11.0%, 13.7%) and summed feature 9 (iso-C_17  :  1_*ω*9*c*/ C_16 : 0_ 10-methyl) (21.1%, 19.2%) (Table 2). To verify the contents of summed feature 9, we further analysed the fatty acid profiles of strains C3-2-a3^T^, B3-2-R+30, and *Luteimonas suaedae* LNNU 24178^T^ using GC-MS. The result from GC-MS assessment indicated that the summed feature 9 (iso-C_17  :  1_*ω*9*c*/ C_16 : 0_ 10-methyl) present in GC detection results was iso-C_17 : 1_* ω*9*c,* which appeared as a peak with RT of 19.990 min. The mass spectrum of iso-C_17 : 1_* ω*9*c* is shown in Fig. 5.

**Table 2.** Fatty acid compositions of strains C3-2-a3^T^, B3-2-R+30 and type strains of related species of *Luteimonas* Strains: 1, C3-2-a3^T^; 2, B3-3-R+30; 3, *L. salinisoli* SJ-92^T^; 4, *L. suaedae* LNNU 24178^T^; 5, *L. endophytica* RD2P54^T^. All the data are from this study. Values are percentages of total fatty acids. −, not detected.

Fatty acids	1	2	3	4	5
Straight-chain					
C_16 : 0_	1.5	0.7	1.1	1.2	1.2
Branched-chain					
iso-C_10 : 0_	0.8	1.7	0.6	1.3	0.5
iso-C_11 : 0_	11.2	12.0	10.0	7.1	5.0
anteiso-C_11 : 0_	0.3	0.5	0.2	0.8	0.1
iso-C_12 : 0_	0.4	0.8	0.3	0.8	0.3
iso -C_13 : 0_	1.4	1.4	1.3	–	0.5
iso -C_14 : 0_	1.3	2.5	2.2	2.9	2.4
iso-C_15 : 1_ F	1.1	1.4	1.0	0.8	0.8
iso-C_15 : 0_	17.1	16.9	22.5	12.4	19.8
anteiso-C_15 : 0_	2.4	2.6	2.5	6.0	2.3
iso- C_16 : 1_ h	0.5	0.9	0.7	2.5	1.4
iso-C_16 : 0_	11.0	13.7	12.7	21.2	18.3
iso -C_17 : 0_	4.7	2.2	5.3	1.2	4.7
iso-C_18 : 0_	0.3	0.2	0.2	0.2	0.3
Hydroxy					
iso -C_11 : 0_ 3-OH	5.7	5.4	4.3	3.3	2.9
iso -C_12 : 0_ 3-OH	0.8	0.9	0.5	1.5	0.5
iso- C_13 : 0_ 3-OH	5.1	3.6	3.1	2.2	3.0
iso -C_15 : 0_ 3-OH	1.1	0.2	0.4	0.7	0.5
iso-C_17 : 0_ 3-OH	0.7	0.4	0.3	0.2	0.1
Unsaturated					
C_15 : 1_* ω*5*c*	1.7	2.9	1.6	1.4	2.7
anteiso -C_17 : 1_* ω*9*c*	0.6	0.4	0.2	1.9	0.6
C_17 : 1_* ω*9*c*	2.3	2.4	2.1	2.2	2.6
Summed features					
1*	0.4	0.4	1.2	1.0	1.4
2*	2.0	1.2	1.1	–	1.0
3*	2.1	1.5	2.4	5.1	4.1
9* (iso-C_17 : 1_*ω*9*c*)	21.1	19.2	20.7	16.0	21.5

*Summed Features are fatty acids that cannot be resolved reliably from another fatty acid using the chromatographic conditions chosen. The MIDI system groups these fatty acids together as one feature with a single percentage of the total.

## Description of *Luteimonas salinilitoris* sp. nov

*Luteimonas salinilitoris* (sa.li.ni.li'to.ris. L. masc. adj. *salinus*, salty; L. neut. n. *litor*, shore, coast; N.L. gen. n. *salinilitoris*, of or coming from a salty shore)

Cells are Gram-stain-negative, aerobic, 0.50–0.53 µm wide and 2.54–3.96 µm long. Colonies are yellow, circular and convex after being cultivated on MA at 30 ℃ for 72 h. Growth is observed at temperatures ranging from 4 to 35℃ (optimum,30℃), in the presence of 0–8.0% (w/v) NaCl (optimum, 6.0%) and at pH levels of 6.0–10.0 (optimum, pH 7.0). The strain is positive for activities of oxidase but negative for catalase activity. Strain is able to hydrolyse Tween 20 but unable to hydrolyse Tween 80, starch, protease and cellulase. In the API ZYM system, it is positive for the activities of alkaline phosphatase, esterase (C4), lipid esterase (C8), leucine arylamidase, valine arylamidase, cystine arylamidase, trypsin, chymotrypsin, acid phosphatase, naphthol-AS-BI-phosphohydrolase, *α*-galactosidase, *β*-galactosidase, *α*-glucosidase, *β*-glucosidase, *N*-acetyl-*β*-glucosaminidase and *α*-mannosidase, but negative for the activities of lipase (C14). In the API 20NE test, it is positive for the activity of aesculin, gelatin hydrolysis and *β*-galactosidase, but negative for the activities of arginine dihydrolase, indole production, nitrate reduction and glucose fermentation. It utilizes dextrin, d-maltose, d-trehalose, d-cellobiose, gentiobiose, turanose, d-raffinose, *α*-d-lactose, melibiose, d-salicylin, *N*-acetyl-d-glucosamine, *N*-acetyl-d-galactosamine, *α*-d-glucose, d-mannose, d-galactose, l-fucose, inositol, gelatin, l-glutamic acid, *β*-methyl-d-glucoside, d-fructose, d-fucose, Tween 40, *β*-hydroxy-d, l-butyric acid, *α*-butanoic acid, acetoacetate and propionic acid as sole carbon sources. The ubiquinone is Q-8. The major cellular fatty acids are iso-C_11 : 0_, iso-C_15 : 0_, iso-C_16 : 0_ and iso-C_17 : 1_* ω*9*c*. The major polar lipids are diphosphatidylglycerol, phosphatidylglycerol, phosphatidylethanolamine and two unidentified phospholipids.

The type strain C3-2-a3^T^ (CGMCC 1.14507^T^ = KCTC 8642^T^), isolated from a soil sample of LungmuCo Lake in Tibet, PR China. The genomic DNA G+C content of type strain is 68.39%. The 16S rRNA gene sequence and genome accession numbers for strains C3-2-a3^T^ and B3-2-R+30 are PQ065621 and PQ151652, JBFWIB000000000 and JBFWIC000000000, respectively.

## Abbreviations

ANI: average nucleotide identity
dDDH: digital DNA–DNA hybridization
GC-MS: gas chromatography-mass spectrometry
Q-8: ubiquinone-8
